# Effect of women empowerment on treatment seeking practice for sexually transmitted infections among women in Ethiopia

**DOI:** 10.1186/s12879-024-09535-2

**Published:** 2024-06-23

**Authors:** Gedefaw Abeje, Meseret Zelalem, Firmaye Bogale, Netsanet Worku

**Affiliations:** 1 College of Medicine and Health Sciences, School of public Health, Department of Reproductive Health and Population studies, Bahir Dar University, Bahir Dar, Ethiopia; 2 Maternal and Child Health Directorate Director, Ministry of Health, Addis Ababa, Ethiopia; 3 Knowledge and Technology transfer, Ethiopian Public Health Institute, Addis Ababa, Ethiopia; 4 Institute of Public Health, University of Gondar, Gondar, Ethiopia

**Keywords:** Ethiopia, Sexually transmitted infection, Treatment seeking practice, Demographic and Health Survey, Women empowerment

## Abstract

**Background:**

Sexually transmitted infections (STI) are public health problems in Ethiopia. Women have a higher chance of acquiring STI. STI complications are more severe in women compared to men. Despite that, treatment seeking for STI among women is poor. Woman empowerment and gender related factors may be playing a role for treatment seeking practice for STI. However, there are no studies that assess the association between these factors and treatment seeking practice for STI among married reproductive age women in Ethiopia. Therefore, this analysis was designed to explore this association in Ethiopia.

**Methods:**

This analysis used the 2016 Ethiopian demographic and health survey (EDHS) data. The 2016 EDHS collected data about STI treatment seeking practice for STI among other variables. Data was analyzed using STATA 17.0. Sampling weights were applied to improve the representativeness of the samples. Descriptive statistics were computed to describe the characteristics of the women. Binary and multivariable logistic regression models were fitted to identify the association between treatment seeking practice for STI and predictor variables. Multicollinearity was checked using variance inflation factors before running the multivariable logistic regression.

**Results:**

In this study, about 28% (95%CI: 20.87, 36.77) married reproductive age women with STI or STI symptoms sought treatment from the formal sector. Women whose husband attended secondary and higher education (AOR, 8.52; 95%CI 1.42, 51.21), and women with higher women empowerment scores (AOR 1.38, 95%CI 1.06, 1.81) had higher odds of treatment seeking for STI or STI symptoms. On the other hand, women who believe wife beating is justified had lower odds (AOR 0.32; 95%CI 0.15, 0.68) of treatment seeking for STI or STI symptoms.

**Conclusions:**

Treatment seeking practice for STI among married reproductive age women in Ethiopia is low. The Ministry of Health and development partners shall conduct further research to identify barriers for treatment seeking practice. Gender variables (women empowerment and belief that wife beating is justified) were significantly associated with STI treatment seeking practice among married reproductive age women. STI prevention and control strategies shall include women empowerment and gender issues as essential component in STI prevention, treatment, and control activities.

## Background

Sexually transmitted infections (STIs) are infections that are transmitted from an infected person to healthy persons through unprotected sexual behavior. Most STIs are caused by chlamydia, gonorrhea, syphilis, trichomoniasis, herpes simplex virus, human papilloma virus and HIV [1]. Sexually transmitted infections are public health concerns, particularly in developing countries. More than 1 million STIs are acquired every day worldwide. The majority of these infections are asymptomatic [1]. Women have a higher chance of acquiring STIs due to their physical structure, and more women tend to be less symptomatic compared to men [2]. Untreated STIs can lead to serious consequences including neurological and cardiovascular disease, infertility, ectopic pregnancy, still births and increased risk of HIV [3].

Sexually transmitted infections are also public health concerns in Ethiopia [4, 5]. An institution based cross-sectional study conducted among pregnant women attending at Tepi University teaching hospital identified high STI prevalence (16.7%) [6]. A study conducted among University of Gondar students estimated that 18.2% of students had a history of STI [7].

Despite the high prevalence, health care seeking behavior for STIs is low. A demographic and health survey (DHS) based data analysis estimated that only one third of women with a history of STI sought care, lower than the case in East African countries [8, 9]. Previous studies identified educational status, occupation, husband’s characteristics, and wealth index as predictors of health care-seeking practice for STIs [8, 9]. Although generally low, the health care seeking behavior for STIs is lower in women compared to men. This situation presents a paradox; the prevalence of STIs is higher among women, and most STI cases among women are asymptomatic. In addition, the complications of untreated STIs are more severe among women compared to men. Therefore, more women compared to men were expected to seek treatment for STI. But this is not true in the Ethiopian context. This scenario gives clues that gender and women empowerment related factors are playing significant role in treatment seeking for STI among married reproductive-age women in Ethiopia. Given the generally low treatment seeking practice for STI, gender related and women empowerment factors will be additional fuel on the impact of STI among women. In this study, women empowerment refers to activities that indicate women’s autonomy and decision making at household level. Studies in other countries reported that women empowerment has positive effect on health care utilization [10, 11]. There are no studies that explore the association between women empowerment and gender related variables with STI treatment seeking practice among women in Ethiopia. Therefore, this study intended to identify the association between women empowerment and gender related factors with treatment seeking practice for STI among married reproductive-age women in Ethiopia based on the 2016 EDHS data.

## Methods

### Data

We used the 2016 EDHS data, which was collected from January 18, 2016, to June 27, 2016, by the Central Statistical Agency (CSA), Ethiopia, with technical assistance from ICF through the DHS. The 2016 EDHS was designed to provide representative data for the country as a whole and for regional states and city administrations of Ethiopia. The sample design was done in two stages. In the first stage, each region was stratified into urban and rural areas, and clusters were selected from both based on the 2007 Ethiopian population and housing census using a probability proportional to size selection. A list of all the households was prepared in all the selected clusters. The second stage of selection used the list of households as a sampling frame and systematically selected a fixed number of households per cluster. Then, all women aged 15–49 who were either permanent residents of the selected households or visitors who stayed in the household the night before the survey were included.

The 2016 EDHS collected data about health including maternal health, child health, family planning, HIV and STI including care seeking practice using structured interviewer administered questionnaire. It collected data on knowledge and attitude of men and women about sexually transmitted diseases and on treatment seeking practice for STI or STI symptoms. In addition, the survey collected information about domestic violence, female genital mutilation, and women empowerment. After permission, we downloaded the Stata format individual (women) data from the DHS program website https://dhsprogram.com/data/available-datasets.cfm.

From a total of 15,683 (weighted) reproductive age women included in the 2016 EDHS data set, 10,014 were married. Only married reproductive age women were included in this analysis because women empowerment variables were collected for married reproductive age women only. From these, women with no history of STI or STI symptoms were excluded from the analysis since the objective of the analysis was to explore the association between gender related and women empowerment factors with treatment seeking practice for STI. A total of 379 (weighted sample) married reproductive age women with history of STI or STI symptoms 12 months before the survey were included to estimate the proportion of women who sought treatment for STI and identify factors associated with treatment seeking for STI.

### Measurements

The outcome variable for this study was treatment seeking practice for STI among married reproductive age women. In the 2016 EDHS data, women were asked if they had history of STI or signs and symptoms of STI 12 months before the survey. Those who reported history of STI or signs and symptoms of STI were further asked whether they sought treatment or not. In this analysis, a woman was considered sought treatment for STI or STI signs and symptoms if she reported she did so from the formal sector (clinics, hospitals, health centers or private doctor).

The predictor variables for this study were socio-demographic variables (age, educational status, wealth index, religion, place of residence and region), women empowerment variables and human right, dignity and safety related variables (age at marriage, belief that wife beating is justified, and experience of sexual or physical violence). A woman empowerment variable, a variable with composite score ranging from 0 to 10, was generated from other variables (employment in the last 12 months, head of sex of the household, ownership of bank account, ownership of a house, ownership of land, ownership of mobile phone, decision maker on health care, decision maker on large household purchase, decision maker to visit family, friends or relatives, a woman can refuse sex and decision maker on contraceptive use). Each response was scored as 0 (representing unfavorable response to women empowerment) and 1 (representing favorable response to women empowerment). Then, we summed the scores to generate the composite score. The higher the composite women empowerment score, the more the woman was empowered.

### Analytical methods

Data analysis was done using Stata 17.0. We recorded the variables with small categories and generated new variables. Sampling weights were applied to consider missing and improve representativeness of the data. Descriptive statistics were computed to describe the characteristics of married reproductive age women. Results are displayed using texts and tables. Proportions were computed for categorial variables. Multicollinearity among predictor variables was checked using variance inflation factors before running the final model. Chi-square test was used to know if treatment seeking for STI was different by woman’s characteristics. Binary logistic regression was done to identify the association between treatment seeking practice for STI and each predictor variable. A multivariable logistic regression model was fitted to identify factors associated with treatment seeking practice for STI among married reproductive age women in Ethiopia.

## Results

### Socio-demographic characteristics

A total of 379 (weighted) married reproductive age women who had STIs or STIs symptoms were included in this study. Majority of the respondents, 299 (78.9%) were rural residents. More than half of the women (55.1%) did not attend formal education. A higher proportion of partners (44%) of women included in this study did not attend formal education as well. About 45% married reproductive age women were Orthodox Christian religion followers and more than one-third, 151 (39.8%), were from the Oromia region. Regarding wealth index, 108 (28.5%) women were in the richest wealth quintile (Table 1).

**Table 1:** Selected socio-demographic characteristics of married reproductive age women with STI or STI symptoms in Ethiopia, 2016 (*N* = 379)

Variable	Number (%)
Age in 5-year groups	
15–19	12(3.2)
20–24	57(14.9)
25–29	82(21.5)
30–34	60(15.8)
35–39	75(19.7)
40–44	73(19.2)
45–49	22(5.8)
Respondent’s education status	
No education	209(55.2)
Primary	117(30.8)
Secondary	21(5.6)
Higher	32(8.5)
Husband/partner’s education status	
No education	167(44.1)
Primary	129(34.0)
Secondary	35(9.3)
Higher	43(11.3)
Don’t know	5(1.4)
Religion	
Orthodox	172(45.5)
Muslim	141(37.3)
Other	65(17.3)
Place of residence	
Urban	81(21.4)
Rural	298(78.6)
Wealth index	
Poorest	71(18.7)
Poorer	71(18.6)
Middle	62(16.4)
Richer	67(17.7)
Richest	108(28.5)

### Women empowerment

Most of the households these women resided (86.7%) were headed by male. Only half of women (54.5%) reported that they were employed at the time of the survey. Similarly, about 50.0% women reported that they cannot refuse sex with their husband (Table 2).

**Table 2:** Women empowerment variables of married reproductive age women with STI symptoms in Ethiopia, 2016 (*N* = 379)

Variable	Number (%)
Employed in the last 12 months	207 (54.5)
Head of the household	
Male Female	329(86.7) 50 (13.3)
Has an account in a bank or other financial institutions	60(15.7)
Own alone or jointly a	
House Mobile telephone Land	234(61.8) 92 (24.2) 199(52.4)
Decide jointly or alone on	
Health care Large purchase To visit family	313(82.5) 304(80.2) 314(82.8)
Can refuse sex	
No Yes	190(50.0) 190(50.0)

### Human dignity, right and safety

Above one third (34.7%) respondents reported that they were married before they were 15 years old. About half of the women with history of STI believe that wife beating is justified if she goes out without telling the husband, if she neglects children and if she argues with her husband. One fifth and two-fifth of the mothers reported that they had experienced sexual and physical violence, respectively in the past 12 months before the survey (Table 3).

**Table 3:** Age at marriage, wife beating and history of violence among married reproductive age women with STI in Ethiopia, 2016 (*N* = 379)

Variable	Number (%)
Age at first marriage	
Less than 15 Between 15 and 18 Greater than 18	132(34.7) 114(30.0) 134(35.2)
Beating is justified if the woman	
Goes out without telling Neglects children Argue with husband Refuses sex Burns food	175(46.1) 197(51.8) 178(47.0) 147(38.8) 159(41.9)
Experienced sexual violence in past 12 months (*N* = 152)	31(20.6)
Experienced physical violence in past 12 months (*N* = 152)	64(41.7)
Respondent circumcised (*N* = 175)	131(75.0)

### Treatment seeking for STI

From a total of 379 reproductive age women with history of STI or STI symptoms, 107 (28.14%; 95%CI 20.87, 36.77) sought treatment for STI or STI symptom from the formal health sector. The Chi square analysis indicated that there was a statistically significant difference in treatment seeking practice for STIs among married reproductive age women by partner education status, place of residence, and wealth index. Similarly, there was statistically significant difference in treatment seeking practice for STI by decision maker on large household purchase and to visit family. Beliefs related to wife beating and history of sexual violence showed a statistically significant difference by treatment seeking practice for STI of married reproductive age women in Ethiopia.

### Factors Associated with Treatment seeking practice for STI

In the bivariable analysis, the woman’s and partner educational status, residence, wealth index, women empowerment score and belief that wife beating is justified were significantly associated with treatment seeking for STI. In the multivariable analysis, partner education status, wealth index, women empowerment score, and belief that wife beating is justified were found associated with treatment seeking for STI among married reproductive age women in Ethiopia (Table 4).

The odds of treatment seeking for STIs for women whose husband attended higher education (above secondary) was about 8 times higher (AOR, 8.52; 95%CI 1.42, 51.21) compared to women whose partner did not attend formal education. Regarding wealth index, the odds of treatment seeking practice for STIs among poorer women was 7.23 (AOR 7.23; 95%CI 1.94, 30.89) times higher compared to women from the poorest wealth index. A unit increase in the woman empowerment score increased the odds of treatment seeking practice for STIs by nearly 40% (AOR 1.38, 95%CI 1.06, 1.81) among married reproductive age women. On the other hand, the odds of treatment seeking practice for STIs among women who believe wife beating is justified is 66% (AOR 0.34; 95%CI 0.15, 0.68) lower compared to women who believe wife beating is not justified (Table 4).

**Table 4:** Factors associated with treatment seeking practice for STI among married, reproductive age women with history of STI or STI symptoms in Ethiopia, 2016

Variable	Treatment for STI		COR (95%CI)	AOR (95%CI)
	Yes n*o* (%)	No n*o* (%)		
Age (in years)				
Less than 25 25–34 Greater than 34	16(23.12) 50(35.16) 41(24.31)	52(76.88) 92(64.84) 128(75.69)	1:00 1.80 (0.78, 4.18) 1.07(0.46, 2.51)	1:00 4.21(0.91, 19.45) 1.83(0.30, 11.04)
Educational status				
No education Primary Secondary Higher	39(18.7) 45(38.3) 10(46.5) 13(41.0)	170(81.3) 72(61.7) 11(53.5) 19(59.0)	1.00 2.70(1.35, 5.39) ** 3.79(1.00, 14.33) * 3.03(0.66, 13.94)	1.00 1.67(0.63, 4.40) 2.49(0.24, 25.66) 0.08(0.01, 0.97) *
Partner educational status				
No education Primary Secondary Higher	32(19.2) 28 [22] 15(43.7) 26(61.1) 5(89.6)	135(80.8) 101(78.0) 20(56.3) 17(38.9) 1(10.4)	1.00 1.19(0.53, 2.65) 3.20(1.25, 8.49) * 6.61(1.58, 27.61) **	1.00 0.65(0.24, 1.71) 1.19(0.32, 4.51) 8.46(1.59, 44.85) *
Place of residence				
Rural Urban	43(53.1) 64(21.4)	38(46.9) 235(78.6)	1.00 0.50(0.09, 2.64)	1.00 0.50(0.09, 2.64)
Wealth index				
Poorest Poorer Middle Richer Richest	8(11.1) 14(19.4) 11(17.0) 22(32.3) 53(48.9)	63(88.9) 57(80.6) 52(83.0) 46(67.7) 55(51.1)	1.00 1.92(0.73, 5.04) 1.63(0.41, 6.42) 3.80(1.36, 10.67) ** 7.62(2.52, 23.04) ***	1.00 7.23 (1.92, 27.13) ** 4.32(0.81, 22.94) 7.96(1.92, 32.92)** 5.63(0.72, 43.94)
Women empowerment score			1.34(1.09, 1.66) **	1.38(1.06, 1.80)*
Age at marriage (in years)				
Less than 15 15–18 Older than 18	37(27.75) 21(18.04) 50(37.12)	95(27.25) 93(81.96) 84(62.88)	1.00 0.57(0.25, 1.32) 1.54(0.77, 3.08)	1.00 0.27(0.11, 0.68) ** 0.68(0.22, 2.04)
Wife beating				
Not justified Justified	62(47.08) 45(18.16)	69(52.92) 203(81.84)	1.00 0.25(0.13, 0.47) ***	1.00 0.34(0.16, 0.74) **
Experienced domestic violence in the last 12 months				
No Yes	86(28.04) 20(28.57)	222(71.96) 51(71.43)	1.00 1.03(0.44, 2.38)	1.00 2.34(0.91, 5.85)

* Significant at *P* < 0.05, ** Significant at *P* < 0.01, *** Significant at *P* < 0.001

## Discussion

STIs are common but treatable public health problems. However many women are not seeking health care for STIs contributing for the increasing burden of untreated infections [12]. Delay or no treatment can allow for the continued transmission and the greater probability of adverse complications [13]. This effect will be the worst among married reproductive age women. This study indicated that only 28.14% (95% CI; 20.87, 36.77) married reproductive age women sought treatment for STI or STI symptoms. Based on this finding and the national target, treatment seeking practice for STI or STI symptoms among married reproductive age women in Ethiopia is low implying the necessity of strengthening the STI prevention and control efforts of the country. The National HIV/AIDS Prevention and Control Strategic Plan targeted to increase the percent of people aged 15–49 with STI treated to 75% by the end of 2027 [14–16]. The finding was similar with a study conducted in Ethiopia [17] but lower than studies conducted in Nigeria [18], Ghana [19], India [20] and East African countries [21]. The possible reason for the difference from the Ghanian finding is the age of the mother. The Ghanian study included only young women (women aged 15–24 years old) where STI prevalence is high. A study from India reported that younger (aged 18–20 years) women were more likely to seek treatment for STI compared to older ones [20].

According to this analysis, women whose partner attended higher education (above secondary) had higher odds of treatment seeking practice for STIs compared to women whose partner did not attend formal education. This finding was in line with studies conducted in Ethiopia [17] and India [22] and a systematic review [23]. The possible reason for this is that husbands with a higher level of education allow their wives to be autonomous and support them in seeking health care services [23]. A study based on DHS data in SSA and Asian countries showed that male education shapes their wife’s health behaviors. According to this study, woman whose partners attended above secondary education were more likely to use contraceptives, attend four or more ANC visits and more likely to deliver their recent baby with the support of a health professional [24]. Similar study in Indonesia supported the fact that partner education is positively associated with maternal health service use [25]. Husbands’ awareness and responsibility as a decision-maker align with the level of education they earn. The better the husband’s education, the greater the possibility of the husband’s support for women’s health seeking practice and the better the husbands understanding of the risk of untreated STIs [25]. In addition to other advantages, allowing all children to attend formal education will improve future women’s treatment seeking practice for STI. Although Ethiopia had a good net enrolment rate (88.7%) for children, only 33.1% progressed to secondary school on 2022 [26].

The odds of treatment seeking practice for STIs among poorer women was 7.23 (AOR 7.75; 95%CI 1.94, 30.89) times higher compared to women with the poorest wealth index. This finding was in line with studies from Sub-Saharan Africa countries [27], East African countries [21], Ghana [22], Nigeria [28], and India [20]. Wealth offers women with STIs the opportunity to afford healthcare seeking and treatment. Wealthy individuals are more likely to pay for their services and women with good economic status are more likely to be able to overcome financial barriers to access health care services. In addition, wealth empowers women and provides them with an energy to make healthcare decisions which is critical to fostering good healthcare-seeking behaviors [27, 29]. Moreover, wealthy people might be more likely to access information through media like radio, internet and television [21, 30] which improves treatment seeking practice.

A unit increase in woman empowerment score increased the odds of treatment seeking practice for STIs among married reproductive age women by nearly 40% (AOR 1.38, 95%CI 1.06, 1.81). This finding was in line with studies in Tanzania [31], and 16 SSA countries [11]. Empowered women might be more autonomous on decision making to seek health care. The women empowerment score contains variables related to decision making. Studies in Ethiopia [32, 33] and SSA countries [34] indicated that the odds of using maternal health services was higher among women who were decision makers compared to women who were not involved in decision making.

The odds of treatment seeking for STIs among women who believe wife beating is justified was 66% (AOR 0.34; 95%CI 0.15, 0.68) lower compared to women who believe wife beating is not justified. This finding was consistent with a DHS based data analysis in Tanzania [31], a study in Ethiopia [33] and a community based study in Bangladesh [35]. A woman’s attitude toward wife-beating is a proxy for perception of her own status. A woman who considers such violence ‘unjustifiable’ is likely to be aware of her greater sense of entitlement, self-esteem, status, and to reflect positively on her sense of empowerment. On the other hand, a woman who considers such violence ‘justifiable’ accepts the right of her husband to control her behavior even by means of violence, which may significantly affect her health seeking behavior [35].

## Conclusions

The proportion of married reproductive age women with history of STI or STI symptoms who sought treatment from the formal sector was low. The Ministry of Health and development partners shall conduct further research to identify barriers for treatment seeking. The higher the woman empowerment score, the higher the odds of seeking treatment for STI. On the other hand, women who had supportive attitude for wife beating had lower odds of seeking treatment for STI or STI symptom. STI prevention and control strategies shall include women empowerment and gender issues as essential component in STI prevention, treatment, and control activities. The ministry of woman and social affairs, in collaboration with development partners, shall explore further why women believe wife beating is justified to reverse the belief.

## Data Availability

The dataset use for this analysis is available on the DHS program repository, available at https://dhsprogram.com/data/dataset_admin/login_main.cfm?CFID=15716167&CFTOKEN=12841bb47e96d711-949548D7-BAFB-A4AB-230A91882FEC6B8E upon permission.

## Abbreviations

AOR: Adjusted Odds Ratio
COR: Crude Odds Ratio
EDHS: Ethiopian Demographic And Health Survey
STI: Sexually Transmitted Infection
CSA: Central Statistics Authority
HIV: Human Immune Deficiency Virus

## References

[1] WHO. Sexually transmitted infections (STIs) 2022 [ https://www.who.int/news-room/fact-sheets/detail/sexually-transmitted-infections-(stis).

[2] Panchanadeswaran S, Johnson SC, Mayer KH, Srikrishnan A, Sivaran S, Zelaya CE (2006). Gender differences in the prevalence of sexually transmitted infections and genital symptoms in an urban setting in southern India. Sex Transm Infect.

[3] WHO. Sexually transmitted infections (STIs) 2024 [ https://www.who.int/health-topics/sexually-transmitted-infections#tab=tab_1.

[4] Plorde DS (1981). Sexually transmitted diseases in Ethiopia. Social factors contributing to their spread and implications for developing countries. Sex Transm Infect.

[5] Gebeyehu S (2022). Magnitude and associated factors for sexually transmitted Disease among Hawassa Industrial Park Workers, Southern Ethiopia. Ethiop J Reproductive Health.

[6] Asres AW, Endalew MM, Mengistu SY. Prevalence and trends of sexually transmitted infections among pregnant women in Mizan Tepi University Teaching Hospital, Southwest Ethiopia: a cross-sectional study. Pan Afr Med J. 2022;42. 10.11604/pamj.2022.42.111.30871 PMC9392006 36033999

[7] Kassie BA, Yenus H, Berhe R, Kassahun EA (2019). Prevalence of sexually transmitted infections and associated factors among the University of Gondar students, Northwest Ethiopia: a cross-sectional study. Reproductive Health.

[8] Shewarega ES, Fentie EA, Asmamaw DB, Negash WD, Fetene SM, Teklu RE (2022). Sexually transmitted infections related care-seeking behavior and associated factors among reproductive age women in East Africa: a multilevel analysis of demographic and health surveys. BMC Public Health.

[9] Handebo S (2020). Sexually transmitted infections related care-seeking behavior and associated factors among reproductive age women in Ethiopia: further analysis of the 2016 demographic and health survey. BMC Womens Health.

[10] Mainuddin A, Begum HA, Rawal LB, Islam A, Islam SS (2015). Women empowerment and its relation with health seeking behavior in Bangladesh. J Family Reproductive Health.

[11] Lewis TP, Ndiaye Y, Manzi F, Kruk ME. Associations between women’s empowerment, care seeking, and quality of malaria care for children: a cross-sectional analysis of demographic and health surveys in 16 sub-saharan African countries. J Global Health. 2022;12. 10.7189/jogh.12.04025 PMC8932460 35356662

[12] Mapp F, Wellings K, Hickson F, Mercer CH (2017). Understanding sexual healthcare seeking behaviour: why a broader research perspective is needed. BMC Health Serv Res.

[13] Ward H, Mertens TE, Thomas C (1997). Health seeking behaviour and the control of sexually transmitted disease. Health Policy Plan.

[14] Tassew SF, Ayenew T, Nega TD, Bantie B, Feleke DG. Knowledge levels of health professional working in Ethiopia toward disaster preparedness, systematic review and meta-analysis. Int J Afr Nurs Sci. 2023:100649.

[15] Ministry of Health. HIV National Strategic Plan: 2023/24-2026/27. 2023.

[16] Afework T, Seid B, Anteneh A, Ayele W, Gebreyesus SH, Endris BS. Burden of mortality from cancer among adults in Addis Ababa, Ethiopia, using verbal autopsy, 2007–2017. Ecancermedicalscience. 2022;16. 10.3332/ecancer.2022.1428 PMC9458272 36158974

[17] Handebo S (2020). Sexually transmitted infections related care-seeking behavior and associated factors among reproductive age women in Ethiopia: further analysis of the 2016 demographic and health survey. BMC Womens Health.

[18] Ebong US, Makinde OA (2021). Determinants of treatment seeking behaviour for sexually transmitted infections in Nigeria. Afr J Reprod Health.

[19] Abuosi AA, Ackon SK, Anaba EA (2022). Health-seeking behaviours of young women with sexually transmitted infections: analysis of the 2014 Ghana Demographic and Health Survey. PLoS ONE.

[20] Puthuchira Ravi R, Athimulam Kulasekaran R. Care seeking behaviour and barriers to accessing services for sexual health problems among women in rural areas of Tamilnadu state in India. Journal of sexually transmitted diseases. 2014;2014. 10.1155/2014/292157 PMC4437401 26316973

[21] Shewarega ES, Fentie EA, Asmamaw DB, Negash WD, Fetene SM, Teklu RE (2022). Sexually transmitted infections related care-seeking behavior and associated factors among reproductive age women in East Africa: a multilevel analysis of demographic and health surveys. BMC Public Health.

[22] Prusty RK, Unisa S (2013). Reproductive tract infections and treatment seeking behavior among married adolescent women 15–19 years in India. Int J MCH AIDS.

[23] Idris IB, Hamis AA, Bukhori ABM, Hoong DCC, Yusop H, Shaharuddin MA-A (2023). Women’s autonomy in healthcare decision making: a systematic review. BMC Womens Health.

[24] Adjiwanou V, Bougma M, LeGrand T (2018). The effect of partners’ education on women’s reproductive and maternal health in developing countries. Soc Sci Med.

[25] Wulandari RD, Laksono AD, Matahari R (2022). Does Husband’s Education Level Matter to Antenatal Care visits? A study on poor households in Indonesia. Indian J Commun Med.

[26] UNICEF, Learning. and Development 2023 [ https://www.unicef.org/ethiopia/learning-and-development#:~:text=Ethiopia%20has%20made%20significant%20progress,making%20it%20to%20secondary%20school.

[27] Seidu A-A, Aboagye RG, Okyere J, Adu C, Aboagye-Mensah R, Ahinkorah BO. Towards the prevention of sexually transmitted infections (STIs): Healthcare-seeking behaviour of women with STIs or STI symptoms in sub-saharan Africa. Sexually Transmitted Infections; 2022. 10.1136/sextrans-2022-055424 PMC10359580 36202610

[28] Mmari KN, Oseni O, Fatusi AO. STI treatment-seeking behaviors among youth in Nigeria: are there gender differences? International Perspectives on Sexual and Reproductive Health. 2010:72 – 9. 10.1363/ipsrh.36.072.10 20663743

[29] Ba DM, Ssentongo P, Musa J, Agbese E, Diakite B, Traore CB (2021). Prevalence and determinants of cervical cancer screening in five sub-saharan African countries: a population-based study. Cancer Epidemiol.

[30] Minyihun A, Tessema ZT. Determinants of access to health care among women in east African countries: a multilevel analysis of recent demographic and health surveys from 2008 to 2017. Risk Manag Healthc Policy. 2020:1803–13. 10.2147/RMHP.S263132 PMC7533273 33061713

[31] Bashemera DR, Nhembo MJ, Benedict G. The role of women’s empowerment in influencing HIV testing. ICF International; 2013.

[32] Wado YD (2018). Women’s autonomy and reproductive health-care-seeking behavior in Ethiopia. Women Health.

[33] Tiruneh FN, Chuang K-Y, Chuang Y-C (2017). Women’s autonomy and maternal healthcare service utilization in Ethiopia. BMC Health Serv Res.

[34] Chol C, Negin J, Agho KE, Cumming RG (2019). Women’s autonomy and utilisation of maternal healthcare services in 31 sub-saharan African countries: results from the demographic and health surveys, 2010–2016. BMJ open.

[35] Khan MN, Islam MM (2018). Women’s attitude towards wife-beating and its relationship with reproductive healthcare seeking behavior: a countrywide population survey in Bangladesh. PLoS ONE.

